# Predictive value of platelet-related parameters combined with pneumonia severity index score for mortality rate of patients with severe pneumonia

**DOI:** 10.4314/ahs.v23i2.22

**Published:** 2023-06

**Authors:** Jing Wang, Lei Cui, Zhengliang Guo

**Affiliations:** 1 The Fourth Hospital of Changsha, Changsha 410006, Hunan Province, China; 2 Department of Critical Care Medicine, Dingzhou People's Hospital, Dingzhou 073000, Hebei Province, China; 3 Department of Geriatrics, Zhuji People's Hospital, Zhuji 311800, Zhejiang Province, China

**Keywords:** Platelet-related parameter, pneumonia severity index score, prediction, severe pneumonia

## Abstract

**Background:**

To analyse the predictive value of platelet-related parameters combined with pneumonia severity index (PSI) score for the mortality rate of patients with severe pneumonia.

**Methods:**

The clinical data of 428 severe pneumonia patients were retrospectively analysed. They were divided into survivor and death groups according to 28-day prognosis. Platelet-related parameters platelet count (PLT), mean platelet volume (MPV), platelet-large-cell ratio (P-LCR) and platelet distribution width (PDW) were measured within 24 hours after admission. The receiver operating characteristic (ROC) curves were plotted. The areas under the ROC curves (AUC) were used to describe the predictive efficiencies of platelet-related parameters, PSI score and their combination for death within 28 days.

**Results:**

On the 28th day, there were 184 deaths and 244 survivors, and the deaths had significantly higher PLT and PSI score but lower PDW, MPV and P-LCR than those of the survivors (P<0.05). The combination of platelet-related parameters and PSI score had the highest sensitivity (96.56%) and specificity (99.34%) and the largest AUC (0.902) for predicting 28-day mortality.

**Conclusion:**

PLT, PDW, MPV and P-LCR are significantly abnormal in patients with severe pneumonia, and the combination of platelet-related parameters with PSI score has the highest predictive value for 28-day mortality.

## Introduction

Severe pneumonia is the most common severe infectious disease in hospitals. With the main symptoms of cough and asthma, it can rapidly lead to mental fatigue, vomiting, pale complexion and other toxic symptoms. The patients with severe pneumonia account for 10-20% of those with nosocomial pneumonia, more than 90% of whom are in the critical state and require treatment in intensive critical unit^1^. Patients with severe pneumonia, if not treated promptly and effectively, will suffer from lung injury, respiratory failure, hepatic and renal dysfunction, systemic inflammatory response syndrome and sepsis, and even multiple organ dysfunction syndrome and death^2^. The mortality rate can be effectively controlled by accurately predicting the efficacy of therapeutic regimen and the prognosis.

At present, the prognosis of patients with severe pneumonia is predicted mostly by the pneumonia severity index (PSI) and Acute Physiology and Chronic Health Evaluation II (APACHE II) scores, but they are subjective and less efficient^3^. Platelets are implicated in inflammatory response and immunoregulation, and can sense pathogens through their own surface receptors and produce immune responses prior to the defense by innate immune cells. They have abnormities in the case of pneumonia and viral hepatitis^4^. In this study, therefore, the predictive efficiencies of platelet-related parameters combined with PSI score for the 28-day mortality risk of patients with severe pneumonia were explored.

## Materials and Methods

### General data

The clinical data of 428 patients with severe pneumonia treated from January 2015 to June 2022 were retrospectively analysed.

**Inclusion criteria:** Patients meeting the diagnostic criteria for pneumonia^5^ with a definite severity^6^ (severe pneumonia: mechanical ventilation or vasoconstrictor drugs for septic shock required, disturbance of consciousness/disorientation, respiratory rate >30 breaths/min, PaO_2_ <60 mmHg, PaO_2_/FiO^2^ <250 mmHg, blood pressure <90/60 mmHg, bilateral or multiple lobe involvement shown in chest X-ray, azotemia, leukopenia, hypothermia, etc.); those with adult community-acquired pneumonia and diagnosed as bacterial infection by sputum specimen or throat swab culture identification; those who received platelet-related parameter detection within 24 hours after admission and were given PSI score; those with complete clinical data.

**Exclusion criteria:** Patients with pneumonia caused by fungi, mycoplasma, chlamydia or viruses; those complicated with other types of diseases affecting platelet-related parameters; those taking anticoagulant or antiplatelet drugs; those complicated with other respiratory diseases (such as respiratory failure and lung cancer); those who died due to non-severe pneumonia; those with mental disorder; those who transferred to another hospital during treatment or voluntarily quit treatment and discharged; those accompanied by severe trauma; those with malignancies.

### Sputum culture and identification

All patients rinsed their mouths with water twice to 3 times in the morning, coughed the first sputum and discarded it. The second sputum was kept in a sterile sputum cup and sent for examination within 2 h. The sputum samples were determined as qualified if there were >25 leukocytes and <10 squamous epithelial cells in each low-power visual field, and subjected to bacterial culture and drug susceptibility test. Bacterial strains were identified by VITEK2 system (bioMérieux, France).

### Detection of platelet-related parameters

Within 24 hours after admission, 5 mL of venous blood was aseptically drawn from each patient, and sent to the laboratory within 1 hour to measure the platelet count (PLT), mean platelet volume (MPV), platelet-large-cell ratio (P-LCR) and platelet distribution width (PDW) using HST-N201 blood cell analyzer (Sysmex, Japan).

### Grading criteria for PSI score

The PSI score was given according to the following grading criteria^7^. Grade I: no predictive standard, and outpatient treatment recommended; grade II: ≤70 points, and outpatient treatment recommended; grade III: 71-90 points, and short-term hospitalization recommended; grade IV: 91-130 points, and hospitalization recommended; grade V: >130 points, and hospitalization recommended. A higher score corresponds to more severe pneumonia.

### Therapeutic regimen

Routine treatment (anti-infection, correction of acid-base balance, nutritional support, respiratory support, prevention and control of complications, etc.) was performed in accordance with the Guidelines for the Diagnosis and Treatment of Community-Acquired Pneumonia^8^.

### Statistical analysis

SPSS 26.0 software was used for statistical analysis. Measurement data were expressed as mean ± standard deviation, and compared by the t-test. Numerical data were compared by the x^2^ test. The receiver operating characteristic (ROC) curves were plotted. The areas under ROC curves (AUC) were calculated to describe the predictive efficiencies of platelet-related parameters, PSI score and their combination for 28-day mortality. P<0.05 was considered statistically significant.

## Results

### Clinical characteristics of patients

The most common clinical characteristic of the 428 patients with severe pneumonia was blood oxygen partial pressure <60 mmHg (accounting for 79.44%), followed by respiratory rate >30 breaths/min (78.27%), pulmonary crackles (70.56%), and cough and expectoration (68.46%) sequentially (Table 1).

**Table 1:** Clinical characteristics of patients

Clinical characteristic	Case No.	Percentage (%)
Blood oxygen partial pressure <60 mmHg	340	79.44
Respiratory rate > 30 times/min	335	78.27
Pulmonary crackles	302	70.56
Cough and expectoration	293	68.46
Body temperature <38°C	255	59.58
Urine output <480 ml/d	139	32.48
Unconsciousness	115	26.87
Blood pressure <90/60 mmHg	115	26.87
Complication with pleural effusion	38	8.88

### Pathogens isolated by sputum cultures

A total of 589 strains of pathogenic bacteria were isolated from the 428 patients, with an isolation rate of 137.62%. Gram-negative bacteria were the main pathogens, accounting for 83.70%. The most common one was *Pseudomonas aeruginosa*, accounting for 30.56%. Besides, the most common Gram-positive bacterium was *Staphylococcus aureus*, accounting for 7.81% (Table 2).

**Table 2:** Pathogens isolated by sputum cultures

Pathogen	Strain No.	Percentage (%)
Gram-negative bacteria	493	83.70
*P. aeruginosa*	180	30.56
*Acinetobacter*	176	29.88
*Klebsiella pneumoniae*	105	17.83
*Escherichia coli*	32	5.43
Gram-positive bacteria	60	10.19
*S. aureus*	46	7.81
*Staphylococcus epidermidis*	14	2.38
Fungus	36	6.11
*Candida albicans*	36	6.11
Total	589	100.00

### Platelet-related parameters and PSI score of deaths and survivors on the 28th day

On the 28 day, there were 184 deaths and 244 survivors, and the deaths had significantly higher PLT and PSI score but lower PDW, MPV and P-LCR than those of the survivors (P<0.05) (Table 3).

**Table 3:** Platelet-related parameters and PSI score of deaths and survivors on the 28th day

	Deaths on the 28th day (n=184)	Survivors on the 28th day (n=244)	*t*	P
PLT (×10^9^/L)	355.57±12.23	270.12±10.35	125.583	<0.001
PDW (fL)	5.02±0.38	9.11±0.41	106.099	<0.001
MPV (fL)	4.12±0.25	6.69±0.37	81.906	<0.001
P-LCR (%)	10.54±1.02	15.79±1.12	50.201	<0.001
PSI score (point)	143.96±4.23	121.32±4.56	52.788	<0.001

### Predictive efficiencies of platelet-related parameters and PSI score for 28-day mortality

**Figure 1:**
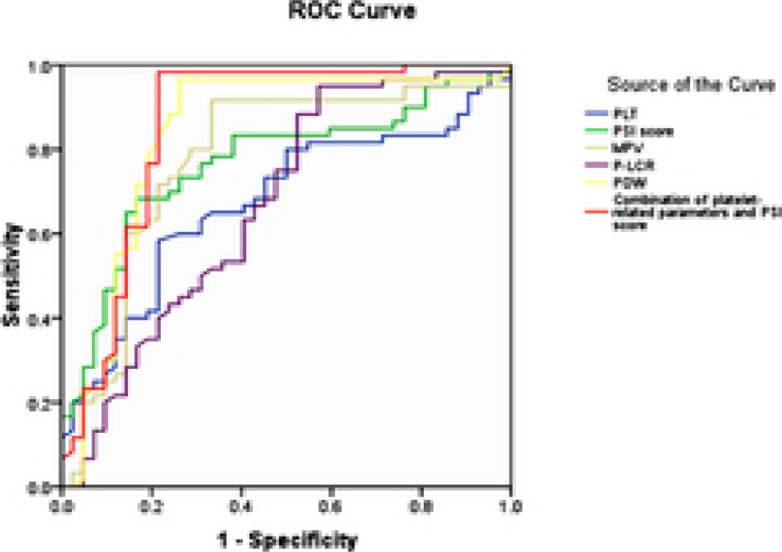
ROC curves of platelet-related parameters and PSI score for predicting 28-day mortality

The optimal cut-off values of PDW, MPV, PSI score, PLT and P-LCR for predicting the 28-day mortality were 6.25fL, 5.08 fL, 136.23 points, 345.12×109/L and 10.87%, respectively, and the AUC values were 0.847, 0.826, 0.779, 0.711 and 0.634, respectively. The combination of platelet-related parameters and PSI score had the highest sensitivity (96.56%) and specificity (99.34%) and the largest AUC (0.902) for predicting the 28-day mortality (Table 4 and Figure 1).

**Table 4:** Predictive efficiencies for 28-day mortality

Parameters	PDW (fL)	MPV (fL)	PSI score	PLT (×10^9^/L)	P-LCR (%)	Combination
Sensitivity (%)	71.34	75.65	81.46	82.04	77.34	96.56
Specificity (%)	78.97	81.32	79.54	78.67	78.41	99.34
95% CI	0.745-0.849	0.723-0.877	0.624-0.811	0.652-0.743	0.554-0.623	0.843-0.945
Youden index	6.25	5.08	136.23	345.12	10.87	-
AUC	0.847	0.826	0.779	0.711	0.634	0.902

## Discussion

Pathogenic microorganism (bacteria, fungi, viruses, etc.) infection, autoimmunity and drug or physicochemical factors are the common causes for severe pneumonia typified by rapid progression and high mortality rate. The mortality rate of patients with severe pneumonia within 28 d after hospitalization is 10-50%^9^-^11^, so effective therapeutic measures are required. Currently, the state of disease and the risk of death are often assessed based on clinical symptoms, laboratory indices and imaging data. However, the routine methods still have obvious limitations. Therefore, it is necessary to explore new methods to improve the predictive efficiency for the 28-day mortality of patients with severe pneumonia, thereby providing novel ideas for ameliorating the prognosis.

Platelets can rapidly produce immune response against the invasion of pathogens to resist pathogenic microorganisms, and also participate in adaptive immunoregulation through T cells, antigen-presenting cells and B cells. Under the combined action of vascular endothelial system and coagulation factors, platelets can also keep the balance between anticoagulation and fibrinolytic systems. Platelet-related parameters PLT, PDW, MPV and P-LCR can reflect the activation degree of platelets. PLT refers to the number of platelets per unit volume of peripheral blood, and reflects the state of platelet production and death in whole blood. Platelets are destroyed after severe infection, producing a large number of inflammatory factors, and enhancing massive platelet aggregation and adhesion at the injury site. Moreover, platelet activation can induce microcirculatory thrombus^12^. PDW mainly reflects the homogeneity of platelet volume. Upon inflammatory stimulus, bone marrow hyperplasia is stimulated to produce new platelets which, however, are generally larger, resulting in poor platelet homogeneity. PDW changes more obviously along with the aggravation of inflammatory response. MPV refers to the mean volume of a single platelet. In the case of infection, platelets are further destroyed and consumed, and new platelets have a larger volume, thus leading to the secondary increase in MPV^13^. As the ratio of large platelets, P-LCR greatly decreases in patients with severe pneumonia due to the existence of many new platelets. Similarly, Fu *et al.^14^* reported that patients with severe pneumonia had higher PLT, and lower PDW, MPV and P-LCR than those of patients with non-severe pneumonia. Hence, there are close associations between platelet-related parameters and inflammation degree upon pneumonia. Taken together, platelet-related parameters can reflect the severity of pneumonia.

In addition, this study showed that the deaths had higher PLT and PSI score but lower PDW, MPV and P-LCR than those of the survivors, demonstrating that they had significantly different platelet-related parameters and PSI score on the 28th day. Qiu *et al.^15^* found that among patients with severe pneumonia, the survivors had a similar initial value of PLT to that of the deaths but a significantly higher final value of PLT. Compared with the deaths, the survivors had higher initial values of MPV and PDW, whereas significantly lower final values. In contrast, we herein only observed the initial values of platelet-related parameters. Possibly, adult patients with bacterial pneumonia were selected as the subjects, while those with bacterial and fungal pneumonia were selected in previous literatures. Besides, the results of ROC curve analysis in this study revealed that the combination of platelet-related parameters and PSI score had high predictive efficiency for the 28-day mortality risk of patients with severe pneumonia, and its sensitivity, specificity and AUC were all significantly superior to those of PDW, MPV, PSI score, PLT and P-LCR. PSI score is often used to predict the 28-day mortality risk of patients with severe pneumonia, but it is highly subjective and had moderate sensitivity and specificity when used alone. Patients with severe pneumonia suffer from vascular endothelial injury and severe hypoxia due to impaired lung function. At the same time, a large of pro-inflammatory cytokines (interleukin-6, C-reactive protein, tumor necrosis factor-α, etc.) are synthesized and secreted upon pathogenic infection. The pro-inflammatory factors lead to platelet activation through multiple pathways, and a larger number of activation products (including prostaglandins, platelet-activating factors and leukotrienes) are secreted, thus enhancing platelet aggregation and adhesion^16^. Moreover, activated platelets can secrete and release cytokines that facilitate the coagulation cascade, thus aggravating the inflammatory response and even inducing thrombosis. As a result, the disease is aggravated, and the risk of disseminated intravascular coagulation, multiple organ failure and death increases. The above-mentioned complications and the risk of death are closely correlated with the degree of platelet activation. Platelet-related parameters PLT, PDW, MPV and P-LCR can reflect the degree of platelet activation that is closely related to inflammatory injury and death risk. Therefore, platelet-related parameters have a higher predictive value for the 28-day mortality risk of patients with severe pneumonia^17^,^18^. Collectively, the patients with more severe pneumonia have more obvious platelet activation, more rapid progression and aggravation of disease, and higher mortality rate. PSI combined with platelet-related parameters can be used to predict the 28-day mortality risk of patients with severe pneumonia.

## Conclusion

Platelet-related parameters have significant abnormalities in patients with severe pneumonia, and the combination of platelet-related parameters and PSI score has higher predictive value for 28-day mortality. This combination is feasibly applicable to the selection of therapeutic regimens, but in-depth studies with larger sample sizes are still required to validate the findings.
